# Acute respiratory distress syndrome emerging after surgical debridement in a patient with extranodal natural killer/T cell lymphoma

**DOI:** 10.1186/s12890-020-01360-y

**Published:** 2021-01-14

**Authors:** Wei Wang, Zhi-Tao Li, Nan-Nan Cui, Guo-Bin Wang, Shui-Qiao Fu

**Affiliations:** Department of Surgical Intensive Care Unit, the First Affiliated Hospital, Zhejiang University School of Medicine, 79 Qingchun Road, Hangzhou, 310003 China

**Keywords:** Extranodal natural killer/t cell lymphoma, Non-hodgkin lymphoma, Acute respiratory distress syndrome, Case report

## Abstract

**Background:**

Extranodal natural killer/T cell lymphoma (ENKL) is a rare subtype of non-Hodgkin lymphoma, and lung involvement is extremely rare. The patients with pulmonary ENKL always presented unspecific symptoms of the respiratory system, such as cough with sputum and varying degrees of fever, while developing into acute respiratory distress (ARDS) was seldomly reported, especially promoted by the surgical procedure.

**Case presentation:**

Here we describe a patient with nasal ENKL and most likely lung dissemination that was regarded as an infection at first. After nonresponse to a period of anti-infective therapy, this patient received surgical debridement. While the histopathology did not show the evidence of infection, but consistent with ENKL. The patient got refractory hypoxemia rapidly after surgery, with the LDH surging to a much higher level than before surgery. The ARDS was diagnosed, and he died on the 5th day after surgery. We postulate that ARDS was due to aggressive lymphoma proliferation promoted by the surgical procedure.

**Conclusions:**

Pulmonary ENKL developing into ARDS was scarce, and was likely attributed to the aggressive tumor cell proliferation after surgery in this case.

## Background

Extranodal natural killer/T cell lymphoma (ENKL) is a rare subtype of non-Hodgkin lymphoma (NHL), which is characterized by aggressive, localized angio-invasion [1]. The most frequently involved site of ENKL at presentation is the upper airway regions, while the disease also could disseminate to various extra nasal sites, such as the skin, gastrointestinal tract, testis, and lymph nodes during its clinical course [2]. The lung is a relatively rare site of involvement in the case of ENKL [3]. The patients with pulmonary ENKL always presented unspecific symptoms of the respiratory system, such as cough with sputum and varying degrees of fever [3], while presentation as ARDS was seldomly reported, especially promoted by the surgical procedure.

Here, we present a case of ENKL with possible lung involvement, which was misdiagnosed as an infectious disease initially and got surgical debridement. The patient developed ARDS and died shortly after surgery, which might be attributed to the aggressive tumor cell proliferation after surgery.

## Case presentation

**Fig. 1:**
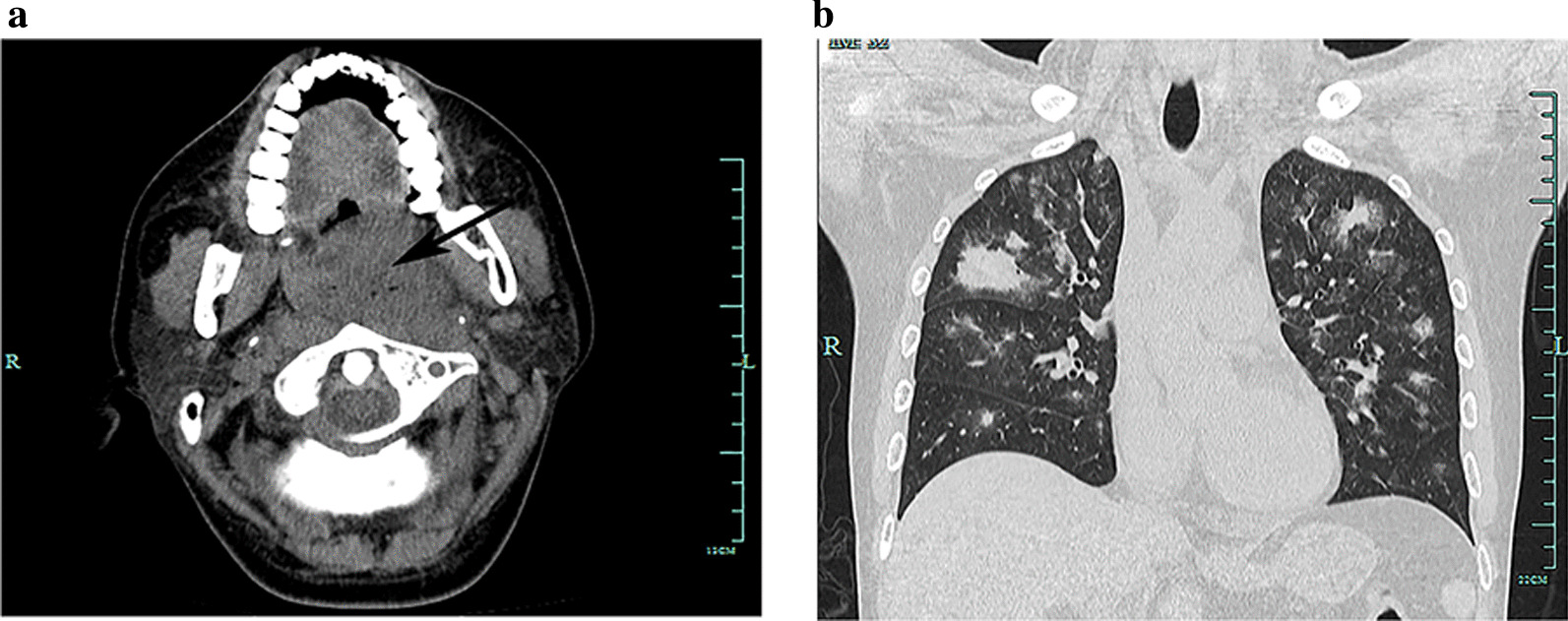
CT images of the neck and the chest on admission. **a** The arrow shows an oval soft tissue mass was in the oropharyngeal cavity, about 3 cm in diameter, with some gas shadows in the lesion. **b** Chest CT revealed multiple lesions among both lungs. Most of them appeared as ground-glass subsolid nodules, some showed consolidation, and the lesion in the right upper lobe showed a mass-like appearance

**Fig. 2:**
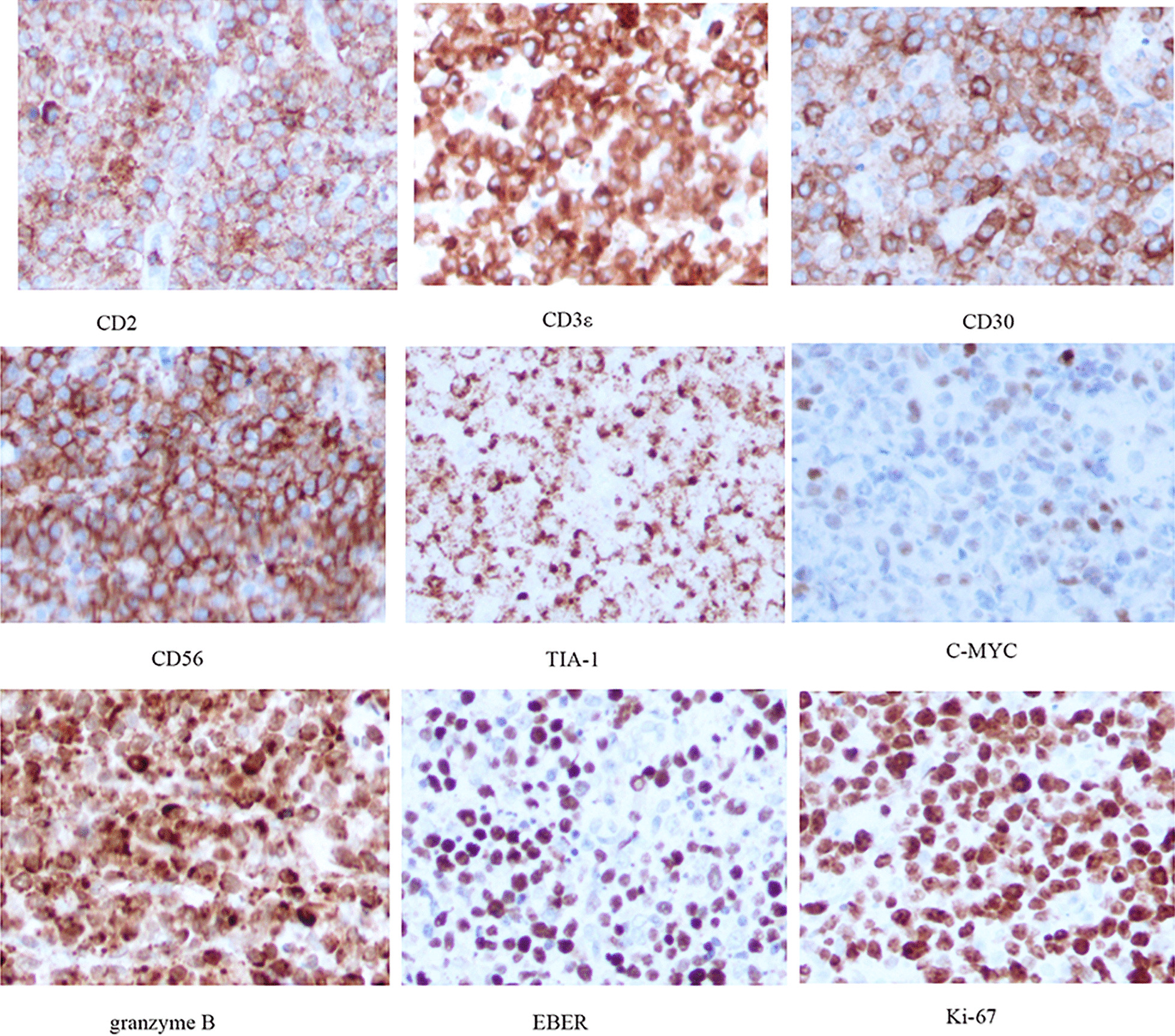
The histopathology of the removed tonsil. Immunohistochemical stains showed positive reactivity for CD-2, CD3, CD30, CD56, c-Myc, TIA-1, and granzyme B. In situ hybridization for Epsteine Barr virus encoded RNA showed positive reaction in atypical cells. The Ki-67 proliferation index showed 80% nuclear staining

**Fig. 3:**
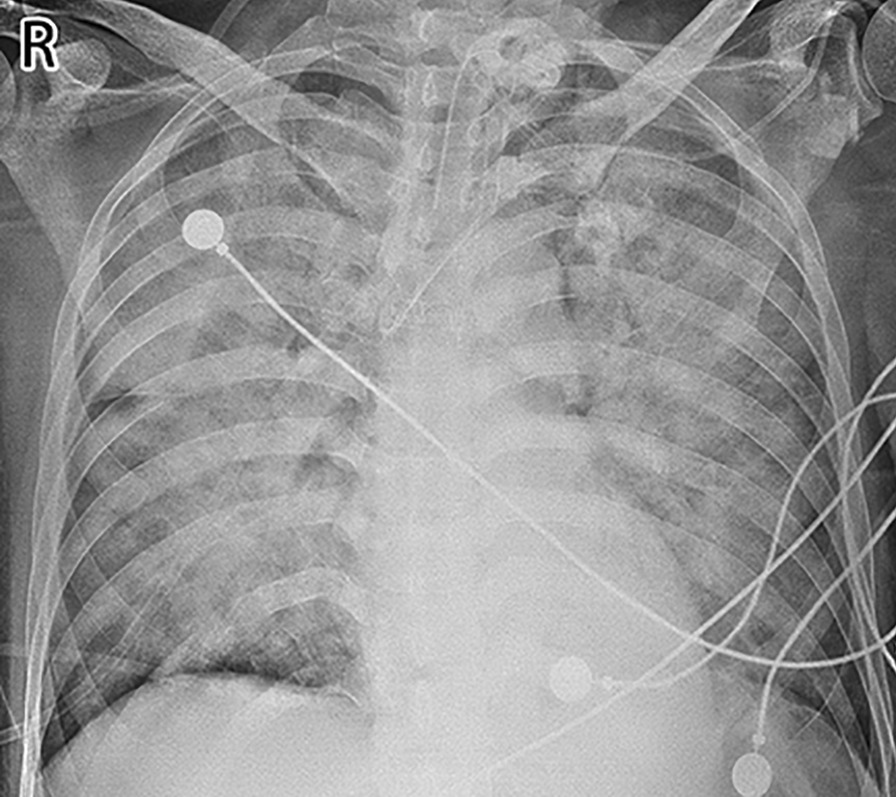
The bedside chest radiograph after surgery. The X-ray found bilateral diffuse opacities in the lung after surgery

A 33-year old man without any medical history was admitted to our hospital because of a sore throat and dry cough for 2 months. He also presented recurrent fever and weight loss of more than 10% in the meantime. The patient was admitted to the infectious department of our hospital. On admission, the physical examination found the left tonsil being grade II swollen, with pus coating the surface. He breathed at 25–30 times per minute; no audible rale was heard on the auscultation. The computed tomography (CT) found an oval soft tissue mass in the oropharyngeal cavity, with some gas shadows in the lesion (Fig. 1a). The pulmonary CT scan found multiple lesions distributed among both lungs. Among them, most appeared as ground-glass subsolid nodules, some showed consolidation, and the lesion in the right upper lobe showed a mass-like appearance (Fig. 1b). The C-reactive protein was 42.37 mg/l (normal range: 0–8 mg/l), the procalcitonin was 0.09 ng/ml (normal range: 0–0.5 ng/ml), the white blood cells count was 4.5 × 10 E9/l (normal range: 4.0–10.0 × 10 E9/l), and the serum lactate dehydrogenase (LDH) was 718 U/l (normal range: 109–245 U/l). Candida albicans were detected by throat swab. The patient was initially diagnosed with the tonsil abscess and pneumonia, which were possibly due to complex infection, including the bacterial and fungal infection. The patient received combined treatment with meropenem, linezolid, and caspofungin for more than a week. However, the initial treatment showed no effect. The results of a series of tests came out, showing that (1,3)-beta-d-glucan (G test) and galactomannan (GM test), sputum culture, acid-fast smear, gomori methenamine silver stain, and respiratory virus screening were all negative. Then the otolaryngologist was consulted, and surgical debridement was performed. During the surgery, a lot of hyperplastic and necrotic tissues were found in the oropharyngeal cavity, and the left tonsil was grade II swollen and festered. The pus and necrotic tissue were debrided, and the left tonsil was taken off. The histopathology found that the tonsil was absent of normal structure, mainly composed of medium-sized cells, with abundant cytoplasm, and the mitotic images can be seen. On immunohistochemical staining, these atypical cells were positive for CD-2, CD3, CD30, CD56, c-Myc, TIA-1, and granzyme B, while CD5, CD10, CD20, CD21, CD23, Bcl-2, Bcl-6, MUM1, PAX-5, CyclinD1, and ALK were negative. Epsteine Barr virus encoded RNA (EBER) in situ hybridization was strongly and diffusely positive in the cells. The Ki-67 proliferation index showed 80% nuclear staining (Fig. 2). The serum EBV DNA level was 1.72 × 10 E6 copies/ml in the test after surgery. These findings were consistent with ENKL. While the patient got refractory hypoxemia immediately upon arrival in the ICU after surgery. The bedside chest radiograph showed bilateral diffuse opacities in the lung (Fig. 3), and ARDS was diagnosed. The CRP increased to 96.16 mg/l, and the PCT increased to 1.09 ng/ml. We continued the experimental anti-infection therapy of meropenem, linezolid, and caspofungin. While following sputum cultures were still negative. Although the CRP and PCT declined gradually to 55.70 mg/l and 0.32 ng/ml respectively, the hypoxemia didn’t resolve. The patient got deterioration rapidly, with the LDH surging to 1660 U/l. He died of ARDS on the 5th day after surgery.

## Discussion and conclusions

The ENKL can be divided into two groups, the nasal ENKL and the extranasal ENKL [4]. In the published series, the nasal ENKL accounts for 60–90% of all cases, with the primary tumor sites located in upper airway regions, including the nasal cavity, nasopharynx, paranasal sinuses, tonsils, hypopharynx, and larynx [4–6]. The extranasal ENKL occurs primarily in extranasal sites (e.g., skin, testis, intestine, muscle), or as a disseminated disease without any apparent nasal involvement [7]. Although bona fide cases of isolated extranasal ENKL exists, extranasal cases might be the dissemination of primary nasal cavity lesions [8].

This patient had lesions in both the upper airway region and the lungs, but we only got the tonsil histopathology result, which has been proved to be the ENKL. We postulate that the lung lesions were more likely to be the pulmonary dissemination of the ENKL than to be pneumonia, since no definite pathogen was found from the admission to the death of the patient. Although the Candida albicans was detected by the throat swab, it is commonly regarded as oral commensal yeast, and negative G test and ineffective caspofungin therapy also supported the Candida albicans was more likely to be commensal than to be invasive. For the same reason, normal PCT tests before surgery and ineffective antibiotics therapy also didn’t support that it was an infection caused by bacteria.

Pulmonary involvement of ENKL is extremely rare [3], and always presented unspecific symptoms of the respiratory system, such as cough with sputum and varying degrees of fever. The radiologic findings can be multiple nodular lesions, mass, consolidation, or atelectasis [3]. As regards this case, the patient presented multiple radiologic findings, as described above.

The prognosis of advanced ENKL is very poor, and the survival at 5 years is approximately 25% [9]. Pulmonary ENKL usually has a fatal outcome [3]. In this case, the patient developed lethal ARDS after surgical debridement, which is seldomly reported before. ARDS is an acute, diffuse, inflammatory form of lung injury that is associated with a variety of etiologies [10]. Among them, sepsis is the most common cause. For this patient, sepsis was suspected initially for increasing PCT and CRP after debridement, and oral symbiotic bacteria were likely to be a source of sepsis. After treatment with powerful anti-infection therapy, the sepsis seemed to be under control, since the PCT and CRP decreased significantly. Paradoxically, the ARDS didn’t resolve, and we saw the LDH surging much higher than before the surgery, which is a signal of tumor cell proliferation. So, we postulated if the ARDS was induced by the aggressive tumor cell proliferation. The fulminant course of aggressive lymphoma presenting as non-infectious ARDS has been rarely reported [11]. And there was once reported that a pulmonary ENKL case presenting as ARDS [12], in which the CT presented as multiple nodular lesions as well.

There is evidence that the growth factors, chemokines, and cytokines after surgery promote tumor growth, invasion, or angiogenesis [13]. In patients with synchronous metastatic disease, resection of the primary may accelerate the growth of the metastatic burden by a variety of mechanisms, including loss of circulating angiogenesis inhibitors produced by the primary [13]. So we postulate that it is the surgical procedure that promoted tumor cell proliferation in the lung, leading to diffusing pulmonary infiltration and the fatal non-infectious ARDS. While the greatest limitation of this case is that we didn’t get the lung biopsy to prove this postulation.

In conclusion, this case describes a patient with nasal ENKL and possible pulmonary dissemination who developed ARDS and died rapidly after surgical debridement. The ARDS was postulated to be due to tumor cell proliferation, which was promoted by the surgical procedure. Therefore, we think in the case of widespread metastasis, we should take care to perform a surgical procedure to the primary lesion to avoid promoting tumor cell proliferation in metastatic sites.

## Data Availability

All data are presented in the manuscript.

## Abbreviations

ENKL: Extranodal natural killer/T cell lymphoma
NHL: Non-Hodgkin lymphoma
ARDS: Acute respiratory distress
CT: Computed tomography
LDH: Lactate dehydrogenase
EBER: Epsteine Barr virus encoded RNA
G test: (1,3)-Beta-d-glucan test
GM test: Galactomannan test
